# Reporting Science and Conflicts of Interest in the Lay Press

**DOI:** 10.1371/journal.pone.0001266

**Published:** 2007-12-05

**Authors:** Daniel M. Cook, Elizabeth A. Boyd, Claudia Grossmann, Lisa A. Bero

**Affiliations:** 1 School of Public Health, University of Nevada, Reno, Nevada, United States of America; 2 Department of Clinical Pharmacy, University of California at San Francisco, San Francisco, California, United States of America; 3 Biomedical Sciences, University of California at San Francisco, San Francisco, California, United States of America; The University of Adelaide, Australia

## Abstract

**Background:**

Forthright reporting of financial ties and conflicts of interest of researchers is associated with public trust in and esteem for the scientific enterprise.

**Methods/Principal Findings:**

We searched Lexis/Nexis Academic News for the top news stories in science published in 2004 and 2005. We conducted a content analysis of 1152 newspaper stories. Funders of the research were identified in 38% of stories, financial ties of the researchers were reported in 11% of stories, and 5% reported financial ties of sources quoted. Of 73 stories not reporting on financial ties, 27% had financial ties publicly disclosed in scholarly journals.

**Conclusions/Significance:**

Because science journalists often did not report conflict of interest information, adherence to gold-standard recommendations for science journalism was low. Journalists work under many different constraints, but nonetheless news reports of scientific research were incomplete, potentially eroding public trust in science.

## Introduction

Despite the rise of electronic media, the print news media continue to provide an important source of science news to the lay public [1], [2]. In fact, because the news media influence public opinion, the press is often used strategically by researchers seeking attention and funding [3], and by advocates seeking policy change [4]. The lay press is also an important source of information on new research for the scientific community [5].

When scientific research is reported in the lay press, important information regarding context and methods is often lost [6]. A study of science news in three leading papers found the omission of research methods to be a steady problem over three decades [7]. Science journalism also often fails to describe the limitations of the study, funding sources supporting the research, or financial conflicts of interests of investigators [2], [8], [9].

Reporting funding sources, financial ties of investigators, and study limitations are important because of potential conflicts of interest. Many scholars agree that the type of conflict of interest most likely to affect the public's trust is a financial conflict where the scientist might gain financially as a result of a particular research outcome [10], [11], [12]. Financial ties of investigators with their sponsors (e.g. stock ownership and consulting income) are associated with the reporting of favorable research results and conclusions for the sponsor [13], [14], [15], [16]. Moreover, financial ties between researchers and their corporate sponsors are increasing in prevalence and magnitude [17], [18]. Biased research can be intentional or unintentional [19], and can result from damaged objectivity at multiple stages in the research process, including conceptualization of the question, design of the research, conduct of the research, or publication (or not) of the research [20]. For these reasons, many scientific journals are now requiring that authors disclose financial ties and potential conflicts of interest [21], [22], [23].

Previous studies of health care journalism do not fully examine the reporting of conflicts of interest, instead focusing on the accuracy of the data being reported. One study of lay press reports assessed 60 health care stories and developed an instrument for scoring the quality of reporting, considering whether the report contains errors of omission in descriptions of the research and methods, or is otherwise misleading about the credibility of sources [24]. Similarly, a review of health news identified “bias and conflicts of interest” as a problem area in reporting, and suggested that readers be told explicitly if researchers and funders could financially benefit from the results [25]. A study of 207 news stories on new drug therapies found that 85 percent cited experts with financial ties to the drug manufacturer, but that only about one-third of these reported the relationship [26]. The few studies to date suggest that there is little reporting on financial conflicts of interest in clinical research.

In addition, few studies have examined how new developments in basic science are reported in the media, with some attention to financial ties. A study of 228 media stories on genetics found that 13% of stories mentioned funding sources for the research and 3% mentioned how the investigator could financially benefit from the discovery [27]. The same research team interviewed scientists and science journalists and found that they mistrust each other greatly, and that one solution would be to regularly but responsibly disclose financial conflicts of interest [28]. Problems of conflicts of interest in science are themselves occasionally the subjects of news stories, usually with negative connotations that fuel a suspicion of science among the public [29]. Forthright discussions of financial ties and conflicts of interest among researchers and funders are associated with public trust and public esteem of the scientific enterprise [30], [31].

Our study examines science journalism covering the most important developments in basic science, clinical research, and engineering from 2004 and 2005. We sought to describe how print media reported new scientific findings over a two-year period, and how funding and financial ties of researchers were reported.

## Methods

Our objective was to determine the extent and nature of reporting of conflicts of interest and research in clinical, engineering, and basic science. We conducted a content analysis of 1152 newspaper stories according to the methods described below.

### Search strategy

We first identified the topics of top news stories in science from the past five years according to year-end lists published in four journals: Discovery, Scientific American, Popular Science, and Science. We excluded topics not relevant to basic science, clinical studies, and engineering, and then took the top fifteen topics for our study. These included global warming, nanotechnology, stem cells, gamma ray, new matter, and ten others (Table 1).

**Table 1:** Frequency of Stories by Topic.

	Frequency	Percent
global warming	109	9.5
toxic exposure	103	8.9
aging	101	8.8
infectious disease	98	8.5
stem cells	98	8.5
gamma ray	89	7.7
reproductive biology	89	7.7
genetics	86	7.5
genomics	86	7.5
nanotechnology	81	7.0
cloning	81	7.0
cancer therapy	75	6.5
genetically modified organisms	42	3.6
new matter	7	.6
chemistry	7	.6
Total	1152	100.0

We searched Lexis/Nexis Academic News, which contains full-text news from a source list of about 260 different United States major and regional newspapers and wire services, for stories on these topics from 2004 and 2005. Some of the topics were overly broad as search terms, so they were paired with the word “study” or “research” in order to find relevant stories describing new studies. We searched “US News” for each of the four regions in the database: Midwest, Northeast, Southeast, and West from January 1, 2004 to November 30, 2005 (when the searching began). This yielded over 9800 hits on news stories. We included news stories from any section but excluded obituaries, book reviews, editorials, and other items not reporting science research. We then conducted stratified sampling by randomly selecting 100 stories from each of the 15 topics, although several topics yielded fewer than 100 hits, and so all the stories were included (Table 1). After eliminating any duplicates, our final sample consisted of 1152 news stories on the top 15 topics in new science and medicine research.

### Data extraction for content analysis of media stories

Three different coders divided the total set of stories. Our coders were an advanced graduate student, a postdoctoral fellow, and an assistant professor. Working from a hard copy of the news item, we entered the information into a qualitative data software package. For each story we noted 20 characteristics: title, author, newspaper, length, section of newspaper, date, topic, category (basic, clinical, engineering), funder of research, financial tie reported for investigators who conducted study, type of financial tie, company with the tie, whose tie, amount of tie, sources quoted, type of financial ties of source quoted, which source with tie, amount of source tie, how the research was portrayed (positive towards the new finding, neutral, critical), and how the financial ties were portrayed. We also had extra fields for research notes and financial tie notes. Any discrepancies were discussed and resolved by the three coders and the principal investigator. Below we report the descriptive statistics from this study.

### Determining concordance between reporting of financial ties in media stories and related scientific journal articles

We determined whether the information on funding sources for the study and financial ties of researchers was readily available to journalists. We conducted a stratified random sample of 1020 newspaper stories that did not report any financial tie information. Using a random number generator, we selected 112 media stories, representing most of our 15 categories. Of these, we were able to obtain the citation information for 73 scientific journal articles that were reported in the 112 stories. We searched PubMed and Google for the scientific journal articles that were mentioned in these media stories. We then examined these journal articles for disclosures of research funding and statements regarding financial ties of the authors.

## Results

Of the 1152 news stories, 56% reported basic scientific research, 38% clinical research, and 6% engineering (Table 2). Of the 15 topics, most concerned global warming, toxic exposures, and aging (Table 1). The average length of the stories was 761 words. The most frequent publishers of the stories were the Associated Press, New York Times, and Washington Post (Table 3).

**Table 2:** Science Category of Sampled News Stories.

Category	Number	Percent
Basic	648	56.3
Clinical	441	38.3
Engineering	63	5.5
Total	1152	100

**Table 3:** Top sources of science news stories (those above 2% of sample)

Newspaper/Source	Number of Stories	Percent of Total
Associated Press	158	13.7
New York Times	68	5.9
Washington Post	53	4.6
Connecticut Post	37	3.2
Boston Globe	33	2.9
Houston Chronicle	27	2.3
San Francisco Chronicle	26	2.3
Ventura County Star	25	2.2
Milwaukee Journal Sentinel	24	2.1

### Funding for the research described in the news story

Funders of the research were identified in 438/1152 (38%) stories, with the most frequent funders being various U.S. government agencies (Table 4). Reported funding sources also included several private nonprofit organizations, a few foreign governments, and over twenty private corporations. The most frequently identified government funder was the National Institutes of Health, followed by the National Aeronautics and Space Administration and the Centers for Disease Control and Prevention. The most frequently identified non-government funders (including the corporate and nonprofit sectors) were the Union of Concerned Scientists, Pfizer, and Advanced Cell Technology.

**Table 4:** Selected Research Funders Identified.

Funder	Frequency	Percent
None Stated	714	61.9%
NIH	49	4.3
NASA	28	2.4
CDC	19	1.6
-------		
Union of Concerned Scientists	9	0.8
Pfizer	4	0.3
Advanced Cell Technology	3	0.3
Aderans	3	0.3

### Financial ties of researchers

Financial ties were defined to include direct corporate sponsorship of research, investigators with stock ownership, equity, patent royalties, consulting fees, honoraria, service on boards of directors or scientific advisory boards, and institutional ties such as the researcher's university having equity in a company funding the research. Financial ties of the researchers who conducted the studies described in the news stories were reported in 11% of stories (132/1152). Researchers were listed with financial ties to major private corporations like Pfizer, Proctor & Gamble, Genentech, Merck, Monsanto, Ford, and others (Table 5). As shown in Table 6, positive identification of ties occurred most often in nanotechnology, cloning, and genomics. Global warming had zero. Of the 132 stories that described financial ties, 65 reported basic science research, 49 reported clinical research, and 18 reported advances in engineering.

**Table 5:** Examples of researcher financial ties to private for-profit corporations.

Pfizer (most frequently found)
Proctor & Gamble
Genentech
Merck
Monsanto
Ford
GlaxoSmithKline
Intel
Wyeth Vaccines
Quantum Dot
Novartis
DuPont
Chiron
Pratt & Whitney
Sygenta

**Table 6:** Financial ties of researchers disclosed by topic.

	Ties Disclosed (no. of stories)	Ties Not Disclosed (no. of stories)
Aging	9	92
Cancer therapy	6	71
Chemistry	0	7
Cloning	23	58
Gamma ray	9	80
Genetics	9	77
Genomics	14	72
Global warming	0	109
GMO	8	34
Infectious diseases	11	87
Nanotechnology	25	56
New matter	0	7
Reproductive biology	12	77
Stem cells	2	96
Toxic exposures	4	99
Total	132	1020

In 105 of the 132 media stories reporting financial ties, we were able to identify the type of financial tie reported. We found various types of financial ties, including research funding, institutional connections, consulting fees, and holding patents and/or selling related products. The type of financial tie reported most frequently in news stories was that quoted researchers were employed by the company that funded the study, often listed as “biotech” and “private lab.” Many of the stories disclosed that the researchers had intellectual property that could create conflicts of interest, such as likely commercial applications and plans to seek patents. Fifty-six stories identified by name the researcher who had the financial tie. In 11 of 132 stories, the dollar amounts of the financial tie were reported, with a wide range. One tie was listed at $24 million, two at $1 million, and two at $100,000.

### Financial ties of sources quoted in stories

We examined how sources quoted in each media story were identified and coded for financial ties of sources, if mentioned. We were able to code the general affiliation of quoted sources in 10% of news items, (111/1152 stories). Sources were most often affiliated with universities (32 stories). The remaining sources reported in stories had various industry affiliations, including specific company names such as Advanced Cell Technology, Alliant, DuPont, Honeywell, and one source was a founder of a company acquired by a competitor of the new research in question. About 5% of the news stories (55/1152, or half of the 111 stories that identified sources) disclosed that the sources quoted had financial ties to the research results, and they named which source had the tie. The dollar amount of the tie was only stated in one instance ($4 million).

### Portrayal of research and financial ties

We found an even split between positive and neutral portrayals of science research, and only a few critical news stories. The research was portrayed positively in about 49% (562/1152) of the media stories, and neutrally in about 49% (560/1152). The remaining 30 news items (2%) were critical of the research. One example of critical portrayal of new research was the news of breakthroughs in animal cloning from August 2005. Several stories presented the new findings together with related ethical dilemmas, the high economic costs, and the doubt surrounding uses of cloned animals [32]. Another story presented the views of environmentalists critical of the methods of new research concerned the results of state testing of toxic vapors in the home, which [33]. An example of a positive story is one on the new cancer drug Herceptin, which quoted patients who think the drug “works wonders,” and is “miraculous” and “revolutionary,” [34]. That story did include the information that the research was funded in part by Roche and Genentech, the makers of Herceptin. In another example of positive portrayal of research, stories about progress in nanotechnology often emphasize the benefits to local economic development and the potential profits from industry partnerships [35]. Neutral stories stated the findings of the study, with no additional qualifying statements.

We also coded for the portrayal of financial ties, although this was more difficult to determine. In the 132 stories that reported a financial tie, 5 were coded as positive, one negative, and 126 were neutral or defied characterization. The story that portrayed the financial tie negatively described research from Dupont on the safety of its own chemical, but also reported that “critics of the company question how the study was done and question Dupont's interpretation of the results,” [36]. Positive portrayals of financial ties often explain that public-private partnerships can be very profitable. One news story describes how the University of New Mexico had joined with a company from Iceland to conduct research in genomics, and stated that the governor and others agreed that the arrangement “was a model for what needs to happen in New Mexico to translate research into commercial products and help expand the state's economy,” [37]. Stories coded as “neutral” portrayals presented the financial tie information without presenting its advantages or disadvantages, or having any other evaluative language.

### Concordance between financial ties disclosed in media stories and scientific papers

We sampled from the 1020 news stories that did *not* report financial ties to determine if the published academic journal article that was covered in the story disclosed that authors or researchers had financial ties. Of the 73 journal articles identified, 20 (27%) reported financial ties of the authors in the published manuscript (i.e., stock ownership, honoraria, consulting), 22 articles (30%) specified that the authors had no competing or conflicting interests, and 31 (43%) had no mention whatsoever of competing interests. Thus, financial tie information was readily available in 44 scientific journal articles which were reported on in stories that did not mention financial ties.

## Discussion

Reporting financial ties of researchers was limited. Because of the importance of high-quality reporting in science, The Commonwealth Fund publishes a “Tipsheet for Reporting on Drugs, Devices and Medical Technologies,” [38]. This is arguably a comprehensive “gold standard” checklist for responsible reporting of new medical therapy, and could be applied to all scientific findings. The Tipsheet recommends that reporters consider seven items: the potential benefits, potential harms, sources of information and their financial ties, strength of evidence, historical context, possible alternatives, and costs related to new treatments. Our study results are relevant to the third “tip,” that reporters should determine the links between sources of information and those who stand to gain from promoting the new research. We found that information about funders, financial ties, and other conflicts of interest seldom appear in the news story, even when the information is clearly available to the journalist.

While the Tipsheet was designed for clinical research news, we found that the reporting of potential conflicts of interest was similarly infrequent across the categories of research; stories about basic science, clinical research, and engineering were all incomplete. New developments in basic science and engineering can have significant impacts on the public similar to clinical research describing new therapies. Furthermore, the vast majority of reporting on new research was favorable or neutral suggesting that readers of the news stories would not be very critical of the research. Yet financial tie information was reported in only about 10% of science news stories. Inclusion of financial tie information in media stories may make readers more skeptical because evidence suggests financial disclosures in scientific articles makes readers more critical of the results [39]. If the sources really had no financial ties, this should also be communicated to readers in order to promote more informed judgment of the science research.

Certainly, journalists face various constraints and barriers while reporting science news. For example, they may confront editorial controls, word limits, time limits and deadlines, and most importantly the facts about sources with financial ties may be difficult to discover. One source of financial tie information about researchers is from scientific journals, but a recent survey found that just 33% of scientific journals have a clear policy on disclosure, with engineering journals having the least disclosure [21]. In our study, we were able to locate financial disclosures in the cited journal articles just 27% of the time. However, even when information on financial ties of researchers was obtainable from the underlying scientific journal article, this information did not make it into the media story. Furthermore, our definition of financial ties was very broad to include research funding. Journals from all disciplines routinely report the funding sources of research studies. Yet funding information, including government and public sources, was reported in less than two-fifths of stories.

Information about the financial ties of researchers is relevant and important to the consumers of the news As more scientific journals adopt financial disclosure standards, information about funding, financial ties and conflicts of interest is becoming increasingly available to the journalists who report the news. We urge science journalists to incorporate the Commonwealth Fund recommendations and to consider such information one important piece of the news story.
